# Low-diversity bacterial microbiota in Southern Ocean representatives of lanternfish genera *Electrona*, *Protomyctophum* and *Gymnoscopelus* (family Myctophidae)

**DOI:** 10.1371/journal.pone.0226159

**Published:** 2019-12-11

**Authors:** Alison Gallet, Philippe Koubbi, Nelly Léger, Mathilde Scheifler, Magdalena Ruiz-Rodriguez, Marcelino T. Suzuki, Yves Desdevises, Sébastien Duperron

**Affiliations:** 1 Muséum National d’Histoire Naturelle, CNRS, Molécules de Communication et Adaptation des Micro-organismes, MCAM, Muséum national d’Histoire naturelle, Paris, France; 2 IFREMER, Channel and North Sea Fisheries Research Unit, Boulogne-sur-Mer, France; 3 UFR 918 « Terre, Environnement, Biodiversité », Sorbonne Université, place Jussieu, Paris, France; 4 Sorbonne Université, Biologie des Organismes et Ecosystèmes Aquatiques BOREA, Paris, France; 5 Sorbonne Université, CNRS, Biologie Intégrative des Organismes Marins, BIOM, Observatoire Océanologique, Banyuls/Mer, France; 6 Sorbonne Université, CNRS, Laboratoire de Biodiversité et Biotechnologies Microbiennes, LBBM Observatoire Océanologique, Banyuls/Mer, France; 7 Institut Universitaire de France, Paris, France; Guangdong Technion Israel Institute of Technology, CHINA

## Abstract

Myctophids are among the most abundant mesopelagic teleost fishes worldwide. They are dominant in the Southern Ocean, an extreme environment where they are important both as consumers of zooplankton as well as food items for larger predators. Various studies have investigated myctophids diet, but no data is yet available regarding their associated microbiota, despite that the significance of bacterial communities to fish health and adaptation is increasingly acknowledged. In order to document microbiota in key fish groups from the Southern Ocean, the bacterial communities associated with the gut, fin, gills and light organs of members of six species within the three myctophid genera *Electrona*, *Protomyctophum* and *Gymnoscopelus* were characterized using a 16S rRNA-based metabarcoding approach. Gut communities display limited diversity of mostly fish-specific lineages likely involved in food processing. Fin and skin communities display diversity levels and compositions resembling more those found in surrounding seawater. Community compositions are similar between genera *Electrona* and *Protomyctophum*, that differ from those found in *Gymnoscopelus* and in water. Low abundances of potentially light-emitting bacteria in light organs support the hypothesis of host production of light. This first description of myctophid-associated microbiota, and among the first on fish from the Southern Ocean, emphasizes the need to extend microbiome research beyond economically-important species, and start addressing ecologically-relevant species.

## Introduction

Microbiota plays multiple fundamental roles in animal biology, including nutrition, immunity, protection and behavior [1]. The study of microbiota in teleost fish is an emerging research topic, initially owing to its relevance to aquaculture and fisheries research [2,3]. Teleosts have also emerged as good models to investigate vertebrate host-symbiont relationships because they are easy to rear, and display relatively limited bacterial diversity compared to other vertebrates, in particular endotherms. Representing half of the vertebrate species, teleosts as a group experience a broad diversity of environmental conditions and life histories, and are thus good candidates to study how microbiota may contribute to host adaptation and resilience [4].

Myctophids are among the most dominant mesopelagic teleost fishes worldwide and are the most dominant in the Southern Ocean [5–7]. An estimated 24 species of Myctophidae strictly occur in the Southern Ocean while 44 more species are occasionally recorded south of the Subtropical Front [6]. They feed on crustaceans, mostly copepods, amphipods and euphausids [8,9], and are important prey items for larger fauna, in particular mammals (seals) and birds (penguins). The Southern Ocean is one of the most extreme marine environments, most notably due to low temperature and isolation from other water masses, resulting in a low diversity of teleosts [6,10]. Teleost adaptation to cold waters has been studied in particular for the endemic Southern Ocean Notothenioidei [10], but very little is known regarding fish-associated microbiota in this environment despite their significance to fish physiology and ecology is well-established [2,3]. To our knowledge, two studies have investigated intestine-associated bacteria using culture-independent methods in four nothothenioid species, in *Chionodraco hamatus* and in *Gymnodraco acuticeps* [11,12]. No study to date has investigated the microbiota associated with the Myctophidae family, and generally very little is known regarding their nutrition. Their diet implies an ability to degrade large amounts of arthropod cuticle, and high levels of chitinolytic activities were indeed measured in the gut from several species, but these originated from the Monterey Bay, and not the Southern Ocean [13]. Interestingly, Myctophidae are referred to as ‘lanternfish’ owing to their production of light. Light emission in metazoans can be of either animal or bacterial origin, the latter through symbiotic interactions [14]. While early works found positive response to bacterial luminescence gene probes, supporting a bacterial origin for this emission, following work invalidated these results and suggested the absence of bacteria-related luciferase genes and activity in Myctophidae [15]. This supports a metazoan origin of light emission, yet the mechanism has not been clearly elucidated and a molecular investigation of bacteria potentially associated to light organs is still lacking.

Given the ecological importance of Myctophidae in the Southern Ocean and general lack of data, this family is a good target group to investigate the composition, organ-specificity and variability of microbiota associated with Southern Ocean fish. In this study, the microbiota associated with species belonging to three genera, namely *Electrona*, *Protomyctophum* and *Gymnoscopelus*, was characterized. The genus *Electrona* is the most numerically abundant [16], and *E*. *antarctica* is the most abundant mesopelagic species endemic to the Southern Ocean [6,17]. Fish were sampled in the region between Crozet and Kerguelen islands where the three genera co-occur. The area is under the influence of three major fronts, the subtropical front, the subantarctic front and the Antarctic polar fronts which all influence myctophid assemblages from the subtropical zone to the subantarctic one and the Antarctic waters [6,18]. A 16S rRNA-based metabarcoding approach was used to identify and compare the bacterial taxa occurring on the gills, the fins, in the luminous organ and in the intestine of fishes. Bacterial communities present in the surrounding water were analyzed and compared with fish microbiota. Altogether, this study provides the first assessment of microbiota composition in Myctophidae species, and one of the first investigation of Antarctic fish microbiota.

## Material and methods

### Sampling

**Fig 1:**
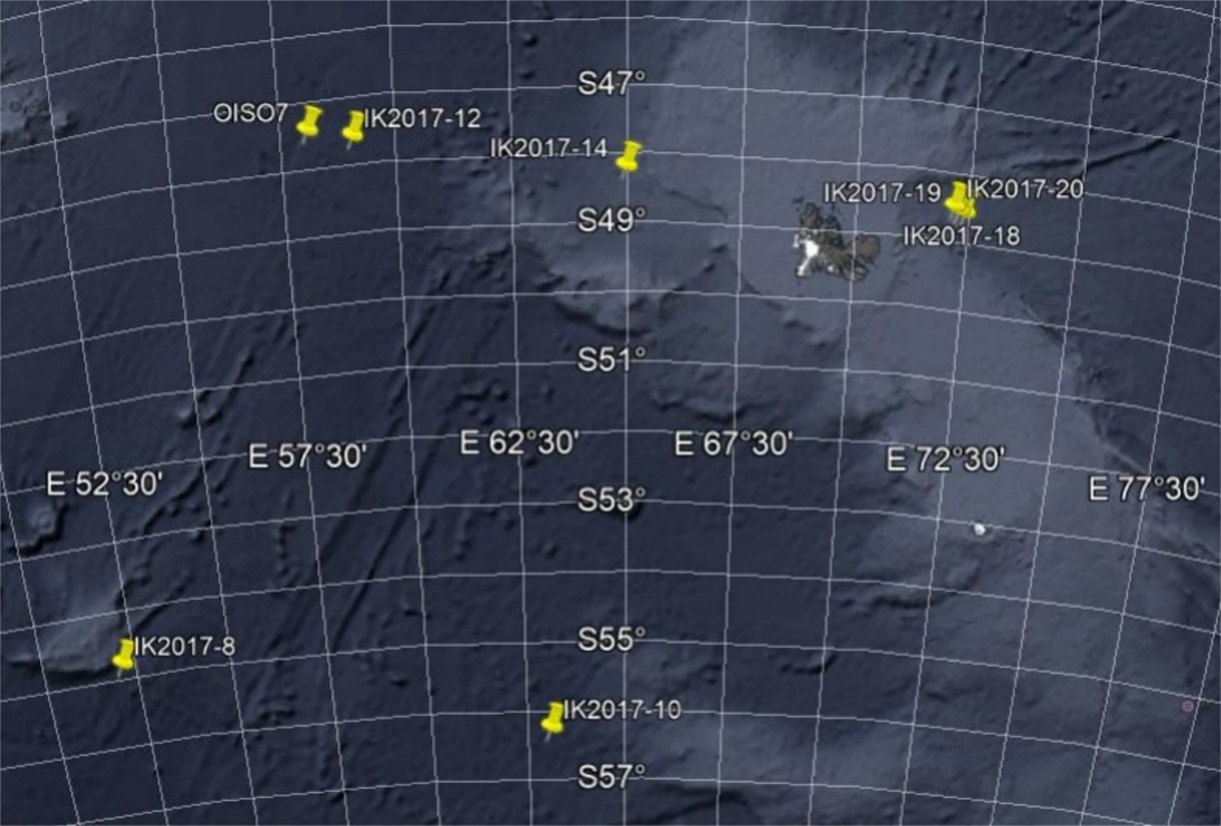
Sampling sites near Kerguelen. Crozet islands are located on the west, not visible. Map obtained using Google Earth.

Samples were acquired within the program VT155 REPCCOAI conducted during cruise MD206/ObsAustral aboard the *RV “*Marion Dufresne”, from 8 stations in the area between Crozet islands and Kerguelen (Table 1 and Fig 1) [19]. Individuals of *Electrona antarctica* (10 specimens), *Protomyctophum bolini* and *P*. *tenisoni* (7 and 4 specimens, respectively), *Gymnoscopelus bolini* and *G*. *braueri* (3 specimens each) were sampled using an IKMT (Isaacs Kidd Midwater Trawl) trawled from the surface to different depths at a speed between 2 to 3 knots (Table 1). The net was 17 m long with a mesh size decreasing from its mouth (4cm) to the cod end where the mesh was 0.5 cm.

**Table 1:** Samples from this study including sample site, coordinates, depth, species, body length and part.

Date	Site	Lat. S	Long. E	Depth (m)	Species	Fish number ID	Shannon index	Length (cm)	Organ	Raw reads	QF, non chimeric reads	Ratio QF/raw (%)	Observed ASVs	Accession	Sample ID
1/14/2017	IK2017-8	54°58.85	51°59.66	60	*Electrona antarctica*	7 –P#11	4.44	4.3	Fin	55886	32354	57.89	56	SAMN12077264	Eant_F1
					*Electrona antarctica*	7 –P#11	4.05	4.3	Gill	40151	24948	62.14	40	SAMN12077265	Eant_Gi1
					*Electrona antarctica*	7 –P#11	1.26	4.3	Gut	75283	38638	51.32	20	SAMN12077266	Eant_Gut1
					*Electrona antarctica*	7 –P#11	2.34	4.3	LO	64024	40648	63.49	31	SAMN12077267	Eant_OL1
					*Electrona antarctica*	8 –P#8	3.82	3.7	Fin	55981	33342	59.56	45	SAMN12077268	Eant_F2
					*Electrona antarctica*	8 –P#8	3.53	3.7	Gill	58216	13669	23.48	36	SAMN12077269	Eant_Gi2
					*Electrona antarctica*	8 –P#8	1.62	3.7	Gut	52072	35848	68.84	7	SAMN12077270	Eant_Gut2
					*Electrona antarctica*	9 –P#10	3.76	4.3	Fin	45203	25126	55.58	41	SAMN12077271	Eant_F3
					*Electrona antarctica*	9 –P#10	3.53	4.3	Gill	36139	18166	50.27	33	SAMN12077272	Eant_Gi3
					*Electrona antarctica*	9 –P#10	1.39	4.3	Gut	39628	6250	15.77	10	SAMN12077273	Eant_Gut3
1/17/2017	IK2017-10	56°27.68	62°58.66	70	*Electrona antarctica*	12 –P#2	3.98	5.6	Fin	28453	2974	10.45	42	SAMN12077274	Eant_F4
					*Electrona antarctica*	13 –P#1	4.67	6.2	Fin	22431	6133	27.34	94	SAMN12077275	Eant_F5
					*Electrona antarctica*	13 –P#1	1.93	6.2	Gill	35501	1749	4.93	15	SAMN12077276	Eant_Gi5
					*Electrona antarctica*	14 –P#4	5.08	4.6	Fin	33881	4917	14.51	81	SAMN12077277	Eant_F6
					*Electrona antarctica*	14 –P#4	0.91	4.6	Gut	39536	24474	61.90	4	SAMN12077278	Eant_Gut6
					*Protomyctophum bolini*	15 –P#51	1.45	5.5	Fin	44925	25090	55.85	42	SAMN12077279	Pbol_F1
					*Protomyctophum bolini*	15 –P#51	0.74	5.5	Gill	60853	45371	74.56	12	SAMN12077280	Pbol_Gi1
					*Protomyctophum bolini*	15 –P#51	0.85	5.5	Gut	50667	35213	69.50	8	SAMN12077281	Pbol_Gut1
					*Protomyctophum bolini*	16 –P#53	1.95	4.9	Fin	34004	19184	56.42	48	SAMN12077283	Pbol_F2
					*Protomyctophum bolini*	16 –P#53	0.78	4.9	Gill	59004	46011	77.98	9	SAMN12077284	Pbol_Gi2
					*Protomyctophum bolini*	16 –P#53	3.41	4.9	LO	85291	1930	2.26	33	SAMN12077285	Pbol_LO2
					*Protomyctophum bolini*	17 –P#50	2.78	5.1	Fin	40350	20005	49.58	65	SAMN12077286	Pbol_F3
					*Protomyctophum bolini*	17 –P#50	0.92	5.1	Gill	58934	43180	73.27	19	SAMN12077287	Pbol_Gi3
					*Protomyctophum bolini*	17 –P#50	1.34	5.1	Gut	53496	38654	72.26	8	SAMN12077288	Pbol_Gut3
					*Protomyctophum bolini*	17 –P#50	2.30	5.1	LO	31776	2338	7.36	19	SAMN12077289	Pbol_LO3
1/19/2017	IK2017-12	47°48.64	58°58.54	530	*Gymnoscopelus braueri*	26 –P#4	0.01	4.7	Fin	58073	33902	58.38	2	SAMN12077320	GGia_F1
					*Gymnoscopelus braueri*	27 –P#5	2.82	4.3	Fin	14028	6676	47.59	22	SAMN12077335	GGia_F2
					*Gymnoscopelus braueri*	27 –P#5	2.99	4.3	Gill	36224	15454	42.66	83	SAMN12077330	GGia_Gi2
					*Electrona antarctica*	28 –P#7	0.01	3.2	Gut	52380	29998	57.27	2	SAMN12077294	Eant_Gut7
					*Protomyctophum tenisoni*	29 –P#32	2.44	4	Fin	4833	1609	33.29	14	SAMN12077314	Pten_F1
					*Protomyctophum tenisoni*	29 –P#32	2.58	4	Gut	34357	8798	25.61	38	SAMN12077315	Pten_Gut1
					*Protomyctophum tenisoni*	29 –P#32	0.90	4	Gill	33862	14430	42.61	9	SAMN12077316	Pten_Gi1
					*Protomyctophum tenisoni*	30 –P#33	1.95	3.2	Fin	42584	11119	26.11	33	SAMN12077317	Pten_F2
					*Protomyctophum tenisoni*	30 –P#33	3.44	3.2	Gill	23270	7994	34.35	36	SAMN12077332	Pten_Gi2
					*Electrona antarctica*	31 –P#8	3.29	3	Fin	32937	5789	17.58	32	SAMN12077302	Eant_F9
					*Electrona antarctica*	31 –P#8	0.48	3	Gut	65710	16983	25.85	3	SAMN12077304	Eant_Gut10
					*Protomyctophum tenisoni*	32 –P#33	0.19	3.1	Fin	45016	31508	69.99	8	SAMN12077334	Pten_F3
					*Protomyctophum tenisoni*	32 –P#33	3.80	3.1	Gill	37021	6989	18.88	69	SAMN12077326	Pten_Gi3
1/19/2017	OISO7	47°40.00	58°00.00	125	Water		4.60			41978	28972	69.02	94	SAMN12077336	Water1
					Water		6.29			99492	61543	61.86	184	SAMN12077337	Water2
1/19/2017	OISO7	47°40.00	58°00.00	600	Water		3.56			43616	28490	65.32	51	SAMN12077338	Water3
					Water		6.32			59862	38712	64.67	205	SAMN12077339	Water4
1/19/2017	OISO7	47°40.00	58°00.00	1000	Water		5.77			63868	41660	65.23	174	SAMN12077340	Water5
1/21/2017	IK2017-14	48°25.22	64°52.26	650	*Protomyctophum bolini*	39 –P#31	4.76	6.3	Fin	26654	5381	20.19	77	SAMN12077296	Pbol_F4
					*Protomyctophum bolini*	39 –P#31	1.65	6.3	Gill	46885	29498	62.92	22	SAMN12077297	Pbol_Gi4
					*Protomyctophum bolini*	40 –P#33	1.09	5.4	Fin	51723	28032	54.20	7	SAMN12077298	Pbol_F5
					*Protomyctophum bolini*	40 –P#33	3.52	5.4	Gill	49545	9151	18.47	47	SAMN12077299	Pbol_Gi5
					*Electrona antarctica*	41 –P#22	3.32	4.9	Fin	25710	7624	29.65	48	SAMN12077312	Eant_F8
					*Electrona antarctica*	41 –P#22	0.05	4.9	Gut	23944	15360	64.15	4	SAMN12077313	Eant_Gut8
					*Protomyctophum bolini*	42 –P#34	2.06	6	Fin	41607	3111	7.48	18	SAMN12077301	Pbol_F6
					*Protomyctophum bolini*	42 –P#34	3.43	6	Gill	42034	7124	16.95	44	SAMN12077305	Pbol_Gi6
					*Protomyctophum bolini*	42 –P#34	2.13	6	LO	8494	2656	31.27	15	SAMN12077307	Pbol_LO6
					*Gymnoscopelus braueri*	43 –P#36	3.60	11.1	Fin	5237	1850	35.33	20	SAMN12077331	GGia_F3
					*Gymnoscopelus braueri*	43 –P#36	5.16	11.1	Gill	41642	7933	19.05	136	SAMN12077324	GGia_Gi3
					*Electrona antarctica*	44 –P#25	2.31	4.5	Fin	53227	17487	32.85	44	SAMN12077321	Eant_F8
					*Electrona antarctica*	44 –P#25	0.40	4.5	Gut	38896	15426	39.66	3	SAMN12077322	Eant_Gut9
1/21/2017	IK2017-14	48°25.22	64°52.26	125	Water		5.52			12669	6673	52.67	93	SAMN12077341	Water6
1/21/2017	IK2017-14	48°25.22	64°52.26	600	Water		6.27			55772	31757	56.94	184	SAMN12077342	Water7
1/21/2017	IK2017-14	48°25.22	64°52.26	1 000	Water		3.66			46454	25059	53.94	56	SAMN12077343	Water8
					Water		5.86			61181	37545	61.37	171	SAMN12077344	Water9
1/25/2017	IK2017-18	48°48.11	72°19.23	900	*Gymnoscopelus bolini*	48 –P#1	3.64	17	Fin	22662	11889	52.46	49	SAMN12077290	Gbol_F1
					*Gymnoscopelus bolini*	48 –P#1	3.61	17	LO	8800	2239	25.44	29	SAMN12077291	Gbol_LO1
					*Protomyctophum tenisoni*	50 –P#9	1.28	3.4	Gut	19765	3622	18.33	15	SAMN12077328	Pten_Gut4
					*Protomyctophum tenisoni*	50 –P#9	1.69	3.4	Gill	42116	16903	40.13	14	SAMN12077329	Pten_Gi4
					*Protomyctophum bolini*	53 –P#10	2.56	3.6	Fin	38022	15931	41.90	44	SAMN12077309	Pbol_F8
					*Protomyctophum bolini*	53 –P#10	2.39	3.6	Gut	36954	19592	53.02	14	SAMN12077310	Pbol_Gut8
					*Protomyctophum bolini*	53 –P#10	0.62	3.6	Gill	37768	18229	48.27	9	SAMN12077311	Pbol_Gi8
1/25/2017	IK2017-18	48°48.11	72°19.23	600	Water		6.09			44181	26783	60.62	181	SAMN12077345	Water10
1/25/2017	IK2017-18	48°48.11	72°19.23	1000	Water		4.68			60145	37872	62.97	116	SAMN12077346	Water11
1/25/2017	IK2017-19	48°46.61	72°11.09	590	*Gymnoscopelus bolini*	52 -P#1	3.92	19.1	Fin	18013	1648	9.15	31	SAMN12077292	Gbol_F2
					*Gymnoscopelus bolini*	52 –P#1	2.33	19.1	Gill	7521	3661	48.68	20	SAMN12077318	Gbol_Gi2
1/26/2017	IK2017-20	48°43.73	72°05.19	190	*Gymnoscopelus bolini*	55 –P#1	3.89	23.6	Gill	24564	8093	32.95	60	SAMN12077323	Gbol_Gi3

F: fin; Gi: Gill; Gut: gut; LO: light organ. Number of raw and quality-filtered (QF) reads are provided. Shannon index and observed ASVs are rarefied to 1,100 reads.

Upon recovery, fish were immediately measured, photographed and dissected using sterile scalpels and tweezers. Caudal fins were sampled, light organs as well as two branchial arcs were dissected. The full intestine (without stomach) was sampled, its content was removed with sterile water pouring to focus on gut-associated communities and avoid bias due to the transient community occurring in the gut contents of different specimens. Samples were frozen immediately in liquid nitrogen then stored at -80°C. Water was sampled from 3 stations, including two where fish were also sampled (IK2017-14 and -18), using Niskin bottles at three depths (125, 600 and 1000 m). Upon recovery, 1L water was filtered on a 0.22 μm nitrocellulose filter and filters were frozen.

No endangered species were harvested for this study. All necessary authorizations and approval of the study protocol were obtained from the “Réserve naturelle des Terres Australes Françaises” (TAAF) prior to the cruise. Réserve nationale naturelle des Terres Australes Françaises” and TAAF administration agreed on the project. The natural reserve is a government board having a committee for environmental protection and a scientific council. PK is a member of the TAAF Scientific Committee.

### DNA extraction and 16S rRNA-based metabarcoding of bacterial communities

DNA was extracted using the QIAGEN Blood and Tissue Kit according to the manufacturer’s instructions (Qiagen, CA), and visualized on an agarose gel. A fragment of the 16S rRNA-encoding gene corresponding to the V4-V5 variable region of *Escherichia coli* was amplified using primers 341F (5’- CCTACGGGNGGCWGCAG-3’) and 805R (5’- GACTACHVGGGTATCTAATCC-3’) [20,21] with Illumina adapters and 8-bp barcodes. The PCR mix contained 1X KAPA2G Fast Ready Mix (Sigma-Aldrich, France), 0.2 μl of each primer (concentration of 0.2 μM), 3.6 μl of ultrapure water and 1 μl of DNA in a final volume of 10 μl. After 3 min of initial denaturation at 95˚C, the PCR was run for 22 cycles (95˚C for 45s, 50˚C for 45s, and 68˚C for 90s), with a final extension step (68˚C for 5 min). Three parallel PCR reactions were run on each sample and then pooled together. PCR products were purified (USB ExoSAP-IT PCR Product Cleanup Kit from Thermofisher, France) and the DNA from different reactions was normalized with the SequalPrep Normalization Plate Kit (96 well, Thermofisher, France), and amplicons were pooled and concentrated by using the Wizard SV Gel and PCR Clean up Kit (Promega, France). Amplicons were sequenced on an Illumina® HiSeq 2500 platform (2×300 paired-end) by FASTERIS SA, Switzerland, in parallel with other projects. Raw reads were deposited into the GENBANK Sequence Read Archive (SRA) database under accession number SAMN12077264 to SAMN12077346, belonging to the BioProject PRJNA531247.

### Sequence analysis

Analysis were performed using the QIIME2 software [22]. Raw reads were demultiplexed, quality checked and trimmed to remove primer regions, paired ends were assembled, chimeric sequences were discarded, and reads were denoised using DADA2 resulting in a list of Amplicon Sequence Variants (ASVs) [23]. Taxonomic affiliations were obtained by the sklearn-based classifier using the SILVA_132_QIIME_release distributed by the Silva project [24]. Sequences matching “Archaea”, “Eukaryota”, “Unassigned”, “Chloroplast” and “Mitochondria”, representing 2.8% of raw reads, were discarded.

Rarefaction curves, alpha and beta diversity indexes were generated using a sampling depth of 1,100 corresponding to the lowest number of quality-filtered reads obtained in a sample. A guide phylogenetic tree was produced to compute UniFrac distances and a principal-coordinates analysis (PCoA) plot based on Weighted UniFrac (WU) dissimilarities was generated [25]. Community richness estimated by Faith’s Phylogenetic Diversity (PD) were compared using Kruskal Wallis tests, and compositions were compared using PERMANOVA. Venn diagrams were drawn using the web-based software available at http://bioinformatics.psb.ugent.be/webtools/Venn/.

## Results

A total of 1,258,379 assembled paired-end bacterial reads were obtained from 61 fish samples (intestine, light organ, fin and gill) and 11 filtered seawater samples (Table 1). These represented 1,683 distinct ASVs. Individual samples yielded between 1,609 and 61,543 reads (mean 18,505). Numbers of reads were sometimes low, leading us to choose a minimal 1,100 reads level for rarefaction-based analyses. At this level, rarefaction curves reached saturation for animal samples at this level, while water samples did not (S1 Fig). It is thus likely that the majority of animal-associated bacterial diversity was successfully captured. At this level, between 2 and 205 (mean 48) ASVs were observed in individual samples (Table 1).

### Diversity and composition of bacterial communities

Water samples displayed highest ASVs diversity with average 137.2±56.3 ASVs, followed by fin and gill samples (mean 40.1±23.6 (24 samples) and 37.5±32.0 (19 samples), respectively; see Table 2 for mean values according to host genus). Light organs displayed on average 25.4±7.9 ASVs (5 samples). Intestine samples displayed markedly lower diversity, with average 10.5±9.9 ASVs (13 samples). Faith’s PD, which accounts for the phylogenetic distance among observed ASVs, was significantly higher in water compared to all fish samples (p<0.001, S1 Table). *Gymnoscopelus* samples displayed a PD comparable to that of water (p = 0.78), while both *Electrona* and *Protomyctophum* samples displayed markedly lower PDs (both p-values versus water <0.001), comparable between them (*Electrona* versus *Protomyctophum* samples, p = 0.62).

**Table 2:** Mean values for Shannon index, and mean observed ASVs and associated standard deviation in each body part (for which sample number exceeds 1) of each genus and in water.

Genus	Organ	Shannon index	Observed ASVs	SD
*Electrona*	Fin	3.85	53.7	20.5
	Gill	3.26	31.0	11.0
	Gut	0.77	6.6	6.8
*Gymnoscopelus*	Fin	2.80	24.8	17.1
	Gill	3.59	74.8	46.7
*Protomyctophum*	Fin	2.12	35.6	24.1
	Gill	1.95	26.4	20.0
	Gut	1.69	16.6	12.4
	LO	2.61	22.3	9.5
Water		5.33	137.2	56.3

Among fish organs, light organs were excluded from alpha diversity comparisons because only 5 samples were analyzed. Fin and gill samples were found to display comparable PDs (p = 0.85), both well above those in intestine samples (both p-values versus intestine <0.001). Sampling date did not affect Faith PD (p = 0.72).

**Fig 2:**
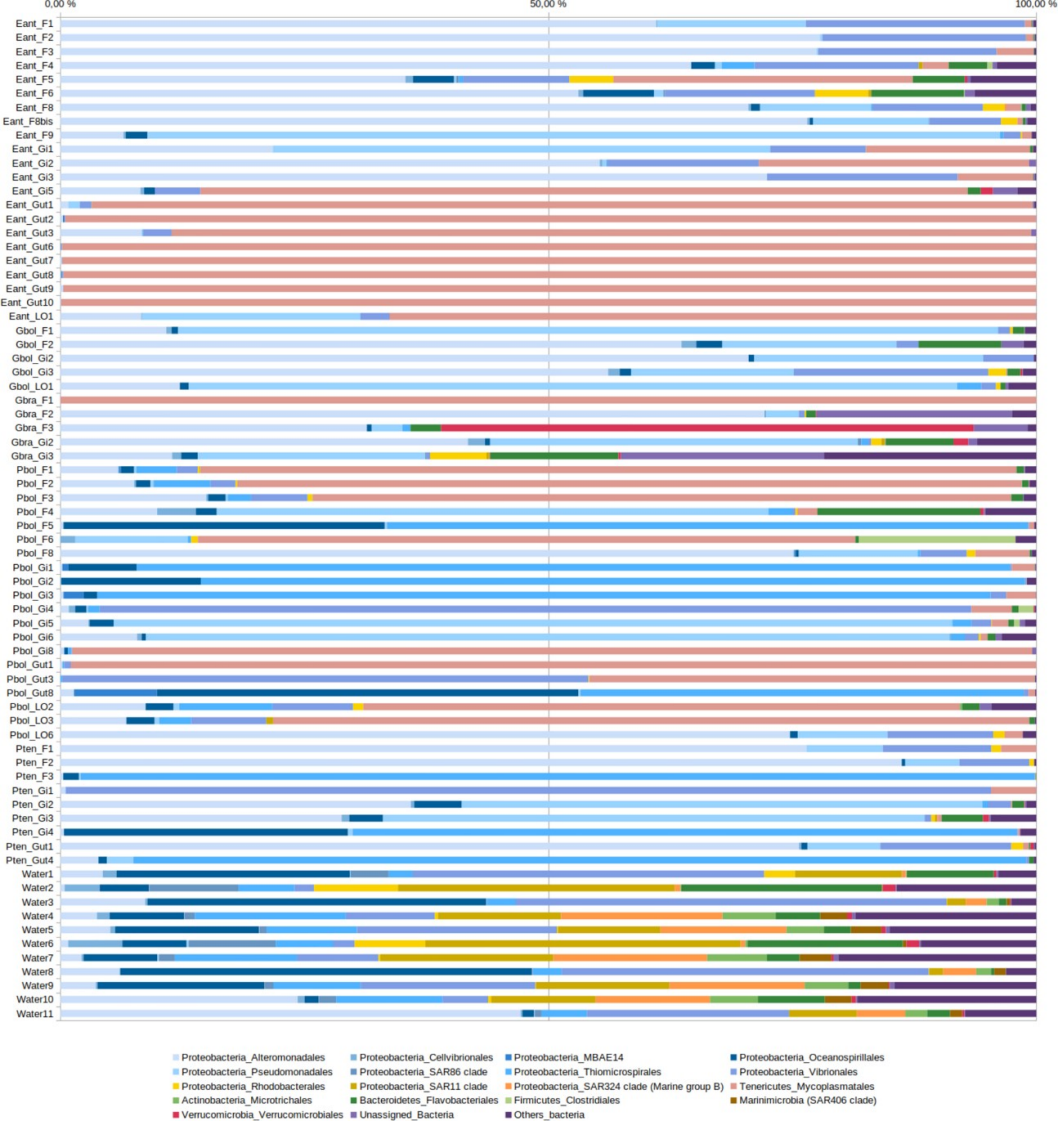
Relative abundances of the different bacterial orders in libraries obtained from the different samples. See Table 1 for nomenclature. Gammaproteobacterial orders are displayed as different shades of blue, alphaproteobacterial orders in shades of yellow. Bacterial orders that are below 3% in all samples are grouped under “Other bacteria”.

The two most abundant bacterial groups in all fish samples were Gammaproteobacteria (notably Alteromonadales, Pseudomonadales, Thiomicrospirales and, to a lesser extent, Vibrionales) and Mollicutes (Mycoplasmatales, Fig 2). In the light organ, dominant ASVs belonged to the Mycoplasmatales (41.3±37.4%, notably genus *Mycoplasma*), Alteromonadales (22.2±29.5%, genus *Pseudoalteromonas*), and Pseudomonadales (22.3±32.8%, genus *Acinetobacter*), and abundances were variable across samples. In intestine samples, Mollicutes were consistently dominant in all samples of *Electrona antarctica* (97.9±4.1% of reads). Mollicutes were also abundant in intestine samples of *Protomyctophum* (mean 29.2±43.6%) but Thiomicrospirales (27.6±40.9%), including two ASVs belonging to the clade SUP-05 of sulfur-oxidizing bacteria, and Vibrionales (13.7±23.1%) represented by two ASVs within genus *Vibrio* present in one sample each, were also abundant in some samples. Despite that several ASVs belonging to the Mollicutes and Gammaproteobacteria were present, a single ASV sometimes represented up to 99% of reads, emphasizing the overall low bacterial diversity in intestine samples (S2 Table). Fin and gill samples displayed dominance of Gammaproteobacteria (68.6±6.2% and 81.6±5.9%, respectively) and Mollicutes (19.1±33.2% and 13.0±27.7%, respectively), with a greater diversity of ASVs compared to other animal sample types. Water samples were dominated by Proteobacteria of the Gamma, Alpha and Delta groups, and Mollicutes represented less than 0.1% of reads in any of the water sample.

### Beta diversity

**Fig 3:**
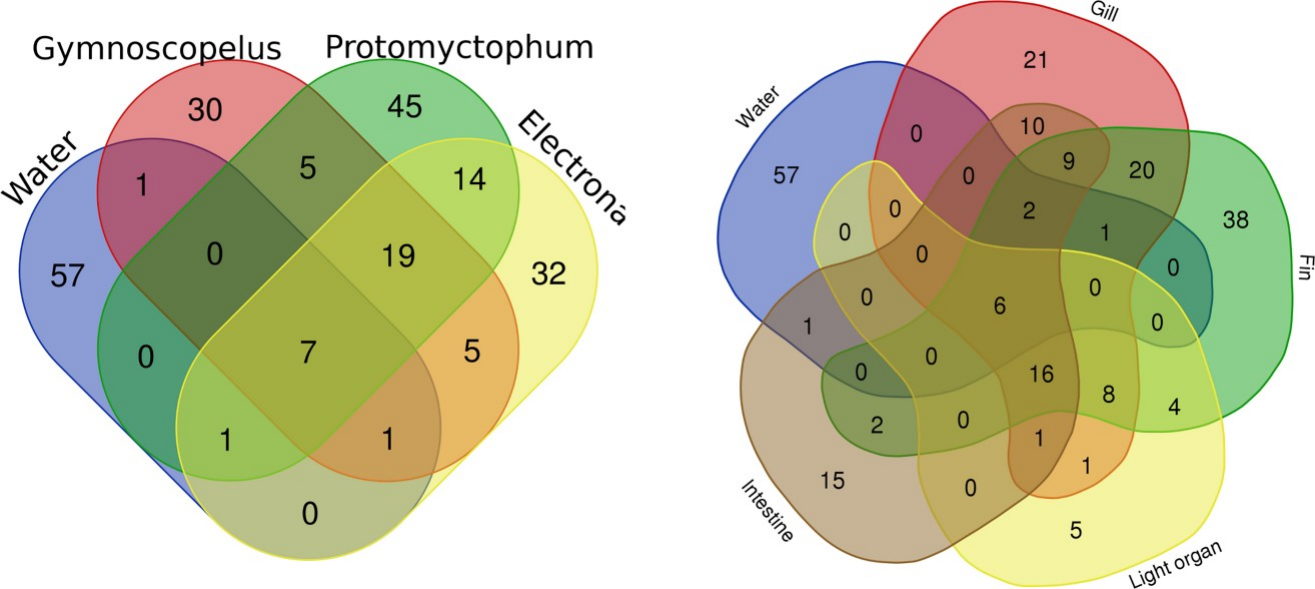
**Venn diagrams displaying the number of shared ASVs among the myctophid genera (left) and organs (right).** Only ASVs representing at least 1% of reads in at least one sample are included.

Among abundant ASVs, *i*.*e*. the 217 representing at least 1% of reads in at least one sample, 53 were shared between at least two genera, or a genus and water. Ten out of the 67 abundant ASVs in water were shared with fish, 7 of which were shared with all three fish genera, and 6 ASVs (all belonging to the Gammaproteobacteria) were shared with all four organ types (Fig 3 left). The three fish genera shared 19 additional ASVs (including 15 Gammaproteobacteria) that were not abundant in water samples. *Protomyctophum* and *Electrona* shared additional 14 ASVs that were absent in *Gymnoscopelus* (11 Gammaproteobacteria and 3 Mollicutes), while the latter genus shared only 5 exclusive ASVs with either of the former two, suggesting a greater similarity between microbiota of *Protomyctophum* and *Electrona*. When comparing organs, 81 ASVs were shared between at least two organs or organ and water. These included the 6 gammaproteobacterial ASVs all organs shared with water, and 16 ASVs that were shared among all organs but absent in water samples (including 11 Gammaproteobacteria and 4 Mollicutes; Fig 3 right).

**Fig 4:**
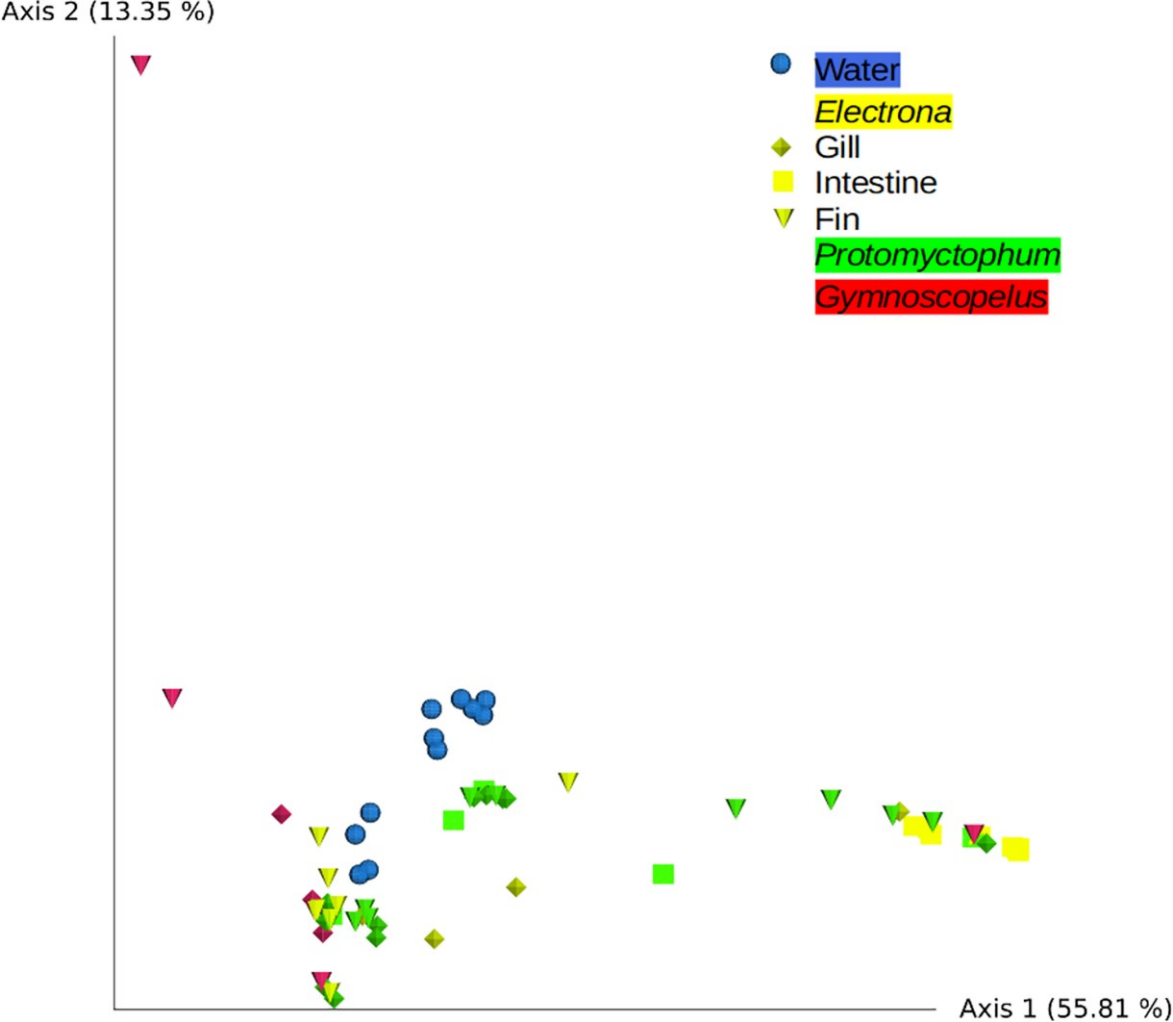
PCoA plot based on WU distances. Colors correspond to water (blue) and genera *Electrona* (yellow), *Gymnoscopelus* (red) and *Protomyctophum* (green); and shapes to gill (diamond), fin (cone), and intestine (square). Light organs as well as two outliers were not included (see text).

In order to compare community compositions, Weighted UniFrac (WU) distances were chosen to account for both phylogenetic proximity among ASVs as well as their relative abundances. Two samples (one fin and one gill) from *G*. *braueri* displayed extremely large distances with all other samples in the exploratory analyses and were removed based on information from further plots and comparisons identifying them as outliers. Water samples formed a tight cluster in the WU PCoA plot compared to fish samples (Fig 4). Samples from the genera *Electrona* and *Protomyctophum* occupied overlapping regions on the graph, while *Gymnoscopelus* samples were spread over the whole graph, suggesting higher variability in the latter (Fig 4).

Statistical comparisons of fish-associated community compositions were performed at the fish genus level. Light organ samples were not included because only 5 samples were available, and n = 1 for two of the genera. PERMANOVA followed by pairwise tests (S1 Table) revealed that communities from *Electrona* and *Protomyctophum* did not display significantly different compositions (p = 0.12), while both differed from water samples (p-values<0.01). *Gymnoscopelus* samples differed from the two other fish genera (both p-values<0.05) and from water (p = 0.002 and p = 0.003, respectively). Unfortunately, no intestine sample was available, and sample composition was highly variable, so the results for *Gymnoscopelus* are to be treated with caution. Community compositions in the different organs were also compared. Gill and fin displayed highly similar compositions (p = 0.77), significantly different from that of water samples (both p-values = 0.001). Intestine-associated bacterial community compositions differed significantly from those of gill, fin and water samples (p-values = 0.001). No significant difference was found in community compositions among the different sampling dates (p = 0.13).

## Discussion

### Low bacterial diversity in the intestine of myctophid fish

Fish body parts displayed markedly lower ASV diversity compared to water samples. Notably, the lowest diversity was observed in the intestine samples, with intermediate values in the fins and gills that are in contact with the environment (see below). Phylogenetic diversity followed the same trend. As the latter accounts for evolutionary distances among ASVs, it can be assumed that higher values suggest a more functionally-diverse community [26]. It is thus likely that lower values in intestine samples reflect a narrower taxonomical and functional diversity compared to fins, gills and light organs, and to water. Lower diversity in the gut versus environment-exposed tissues is commonly reported in vertebrate microbiome studies, possibly because of the occurrence of digestive enzymes, and low pH which make for a more stressful habitat, although fish guts displays higher oxygen levels than endotherm guts [11].

Whether the alpha diversity levels observed here and in Southern Ocean fishes in general are overall lower than in fishes from less extreme (*e*.*g*. warmer) environments is hard to say at this stage because of the lack of data in this region. A study based on clone libraries (~500 clones analyzed) for example revealed only 17 and 6 OTUs in the gut of notothenioid fishes *Notothenia coriiceps* and *Chaenocephalus aceratus*, respectively, with dominance of gammaproteobacterial genera *Photobacterium*, *Vibrio* and *Aliivibrio* and estimated coverage above 96% [11]. Sedlacek isolated 38 strains of *Enterobacter* and 6 of *Aeromonas* from 4 Notothenioidei fish species but did not provide any estimate of their relative abundance [27]. A recent study on 4 species (*Trematomus bernacchii* and *Pagothenia borchgrevinki* (family Nototheniidae), *Chionodraco hamatus*, (family Channichthyidae) and *Gymnodraco acuticeps* (family Bathydraconidae) analyzed the gut content microbiota and showed a predominance of Proteobacteria, Actinobacteria and Firmicutes, yielding several hundred OTUs per sample (yet without exact numbers provided [12]).

Most previous studies, including those discussed above, have used Operational Taxonomic Units (OTUs) for sequence reads clustering instead of ASVs. ASVs have emerged recently as a more appropriate mean of evaluating microbial diversity [23], but on the other hand diversity metrics inferred from the two approaches are hard to directly compare. A recent work in our group on laboratory-reared medaka *Oryzias latipes* using a similar approach revealed an average of 95 ASVs in gut samples, suggesting that myctophid fish from the present study display a much lower bacterial diversity in their intestine [28]. In a recent analysis of rainbow trouts (*Oncorhynchus mykiss)* in an aquaculture setting, between 15 and 29 ASVs were identified in the gut, slightly above values reported here, with a dominance of Mycoplasmatales as found here [29]. The paucity of literature regarding Southern Ocean fish microbiota does not allow direct comparison, and it is possible that a fraction of the diversity was missed using our approach due to relatively low total numbers of reads in certain samples, yet overall it seems that myctophids from this study display a low diversity of bacteria in their intestine compared to other fish in which comparable analyses were conducted.

### The gill and fins display specific bacterial communities at the interface between fish and seawater

The intestine-associated bacterial community differed from that of the seawater, but interestingly, also from that of gill and fins, as previously reported in the rainbow trout [30]. The latter two, dominated by Gammaproteobacteria and Mollicutes, were also significantly different from seawater communities, emphasizing the peculiarities of surface epithelia and associated mucus, sit at the interface between seawater and hosts [29,31]. Proteobacteria are reported as dominant members of the gill and skin-associated communities of numerous teleosts, including for example the seabass *Dicentrarchus labrax* and seabream *Sparus aurata* [2,32]. In the present study, we analyzed fins instead of fish skin because the latter was often damaged during fishing operations. Although skin and fins likely represent similar habitats, some work has shown that differences could exist between associated communities that may reflect stochastic effects. Nevertheless, in one study, Proteobacteria were found dominant in both fin as well as skin samples of *D*. *labrax* and *S*. *aurata* [33]. The mucus covering gill and fins surfaces has a protective role, and acts as a filter and first line of defense against parasites and pathogens. However, the abundance of organic substrates available to heterotrophic microorganisms may on the other hand attract various microorganisms not necessarily abundant in surrounding water, and is, to a certain extent permissive to bacterial colonization, allowing a diversity of bacteria to establish [31]. Brown *et al*. reported 50 to 57 ASVs in the gills of *Oncorhynchus mykiss*, with dominance of Proteobacteria, in the range of values reported here [29], while reports on the seabass and seabream recently indicated higher diversity (457 to 539 ASVs, with 2 to 24 belonging to the core microbiota) [32]. Bacterial diversity thus seems to vary greatly among species, and it is hard to conclude whether the diversity level observed in Myctophidae gills and fins should be considered low.

### Significance of microbiota composition to myctophid biology and ecology

Dominant members of fish-associated communities included various Mycoplasmataceae (Mollicutes), while none was abundant in water samples. Mycoplasmataceae are commonly reported as abundant in fish microbiota studies, notably in several omnivorous (e.g. *Gillichthys mirabilis* and *Lagodon rhomboides)* and carnivorous species (e.g. *Salmo salar*, *Sciaenops ocellatus*) [3]. They for example dominate in all salmonid species investigated to date, including wild and farmed [29,30,34]. In these, an antagonism apparently exists between *Mycoplasma* and pathogenic *Vibrio*, suggesting that the former prevents the establishment of the pathogen [35]. *Mycoplasma* are also dominant in the gut of farmed rainbow trouts in which they are likely fermenting various substrates and contributing the host lactic and acetic acid [36]. Interestingly, *Mycoplasma* were most abundant in trout fed with insect-enriched diets, and chitin was suggested as a prebiotic. Similarly, the arthropod-based, chitin-rich diet of Myctophidae may favor dominance of *Mycoplasma* in specimens from our study. Overall, for these reasons, it can be hypothesized that *Mycoplasma* could be beneficial partners in a long-established symbiosis with fish guts.

Interestingly, members of the Vibrionales and the genus *Vibrio*, while usually important members of fish microbiota with diverse functions ranging from pathogenic to probiotic [3], were not abundant in fish samples, except in a few samples of *Protomyctophum*, and were almost completely absent in *Electrona* samples. This is congruent with the aforementioned antagonism hypothesis, and suggests a pathogenic status for identified Vibrionales ASVs, with high abundances corresponding to infected individuals. The presence of members of the SUP-05 cluster in the intestine, gill and fins of several specimens of *Protomyctophum*, with sometimes high abundances, is also intriguing. This bacterial clade indeed includes mostly aerobic, sulfur-oxidizing autotrophic bacteria, some members being symbiotic with deep-sea metazoans from seeps and vents [37].

Addressing the role of microbiota for Myctophidae is thus not straightforward at this stage. Regarding nutrition, Myctophidae consume mainly copepods, euphausiids, pteropods and hyperiids, and some non-Antarctic species within this family display high levels of chitinolytic enzymes [13]. Pakhomov pointed no substantial differences in the regime, based on prey availability rather than selection, among 36 species including three from the present study [8]. Recently however, some level of trophic niche differenciation was suggested among members of the genera *Electrona*, *Gymnoscopelus* and *Protomyctophum* sampled from around the Kerguelen Islands, including all 5 species from the present study [5]. *Gymnoscopelus bolini* and *G*. *braueri* for example displayed higher δ^15^N signatures, indicative of higher trophic level compared to *Electrona antarctica* and *Protomyctophum* species. Interestingly, *Gymnoscopelus*-associated bacterial communities tend to differ from those in *Electrona* and *Protomyctophum* here, however at this stage the link between microbiota composition and trophic level cannot be tested. Another trait that may involve bacteria is bioluminescence [14]. Myctophid fish are indeed known to emit bioluminescence in their ventral and lateral light organs. While early works suggested a bacterial origin for this emission, following work rather pointed towards the metazoan origin of the process [38,39]. We specifically investigated for the presence of ASVs corresponding to two reportedly light-producing bacterial genera, *Aliivibrio* and *Photobacterium* (8 and 1 ASVs, respectively) in light organ samples of *Gymnoscopelus* and *Protomyctophum*. None was above 2.5% of reads, supporting previous reports that indicate that bioluminescence in this teleost family is not due to a bacterial symbiosis [15,39].

In conclusion, this study shows that Gammaproteobacteria and Mollicutes are the dominant bacterial taxa present in the intestine, fin, gill and light organs of three genera of plankton-feeding Myctophidae. *Electrona*, the most abundant genus in the Southern Ocean, and *Protomyctophum* display overall similar microbiota compositions while that of *Gymnoscopelus* is apparently different but warrants further study because of the low number of samples included in this study. The intestine-associated microbiota displays low diversity and is different from that of gills and fins, with a dominance of Mollicutes in particular in *Electrona*. The overall rarity of Vibrionales is to be noted, as is the occurrence of members of the clade SUP-05 in *Protomyctophum*. Potential light-emitting bacteria were almost absent from light organs. These findings provide the first assessment of microbiota composition in the most abundant fish family from the Southern Ocean. However, in this study, sampling was performed over a short period of time, and the existence of seasonal variation in microbiota composition should be further monitored throughout the year. To test whether low diversity is a specificity of the family Myctophidae or is due to their extreme Southern Ocean habitat, it will be necessary to perform similar analyses on species that are not endemic to the Southern Ocean. Finally, the next step will be to investigate the roles of Myctophidae microbiota using functional approaches, in order to evaluate the role of microbiota in host biology and nutrition.

## Supporting information

S1 Fig Rarefaction curves by sample type, indicating that samples of animal origin (gill, fin, intestine and luminous organ) reach saturation at the selected 1,100 sequencing depth. Water samples do not reach saturation and associated diversity is thus likely underestimated. (TIFF)

S1 Table Results from Kruskal-Wallis tests done on Faith Phylogenetic Diversity (sheet 1) and PERMANOVA analyses (sheet 2) conducted to test the effect of genus, tissue and date, and associated pairwise tests. P-values < 0.05 are in bold.
(XLSX)

S2 Table Summary of identified ASVs and their percentage occurrence in each individual sample; maximum observed percentage, taxonomic affiliation and sequence are provided. (XLSX)
