# Supplementary figures and images for: Low-diversity bacterial microbiota in Southern Ocean representatives of lanternfish genera Electrona, Protomyctophum and Gymnoscopelus (family Myctophidae)
Source: PLoS One. 2019 Dec 11;14(12):e0226159. doi: 10.1371/journal.pone.0226159 (PMC6905552; doi:10.1371/journal.pone.0226159)

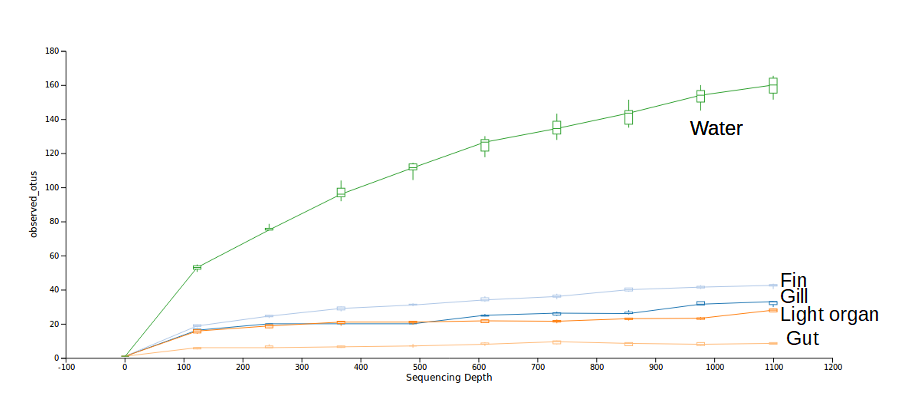

Supplement: S1 Fig — (TIFF) [file pone.0226159.s001.tiff]
